# β-Phase Morphology in Ordered Poly(9,9-dioctylfluorene) Nanopillars by Template Wetting Method

**DOI:** 10.1007/s11671-010-9788-6

**Published:** 2010-09-26

**Authors:** R Palacios, P Formentin, E Martinez-Ferrero, J Pallarès, LF Marsal

**Affiliations:** 1 Departament d'Enginyeria Electrónica, Eléctrica i Automática, Universitat Rovira i Virgili, Avda. Països Catalans 26, 43007 Tarragona, Spain; 2 Institute of Chemical Research of Catalonia (ICIQ), Avda. Països Catalans 16, 43007 Tarragona, Spain

**Keywords:** Template wetting, Nanoporous alumina, PFO, Nanopillars, Luminescence, Raman spectroscopy

## Abstract

An efficient method based in template wetting is applied for fabrication of ordered Poly(9,9-dioctylfluorene) (PFO) nanopillars with β-phase morphology. In this process, nanoporous alumina obtained by anodization process is used as template. PFO nanostructures are prepared under ambient conditions via infiltration of the polymeric solution into the pores of the alumina with an average pore diameter of 225 nm and a pore depth of 500 nm. The geometric features of the resulting structures are characterized with environmental scanning electron microscopy (ESEM), luminescence fluorimeter (PL) and micro μ-X-ray diffractometer (μ-XRD). The characterization demonstrates the β-phase of the PFO in the nanopillars fabricated. Furthermore, the PFO nanopillars are characterized by Raman spectroscopy to study the polymer conformation. These ordered nanostructures can be used in optoelectronic applications such as polymer light-emitting diodes, sensors and organic solar cells.

## Introduction

Polyfluorene-type-π-conjugated polymers have recently received a lot of interest as active materials due to their charge transport properties, high quantum yield and chemically tuneable emission wavelength [1,2]. Poly(9,9-dioctylfluorene) (PFO) is the most promising candidate as blue-light-emitting polymer because of its highly efficient photoluminescence, good thermal and chemical stability at elevated temperatures and solubility in organic solvents. In addition, the polymorphism of PFO provides to study the influence of phase morphology on the photophysics of it without chemical modifications. Four different phases of this material may exist, which can be identified by spectral features apparent in absorption and photoluminescence. A well-studied example is the β-phase, which is a phase with a planar zigzag conformation resulting in an extended conjugation length. Recently, β-phase PFO has been shown as a favourable environment for charge carriers, with an enhanced charge carrier mobility [3]. These properties make it a good material for organic display devices. β-Phase morphology may be induced by different experimental process [4,5] and the incorporation of it into polyfluorene nanostructures could be an interest advanced in order to exploit the benefits of this morphology in polymer structures based in photonic and electronic devices.

Organic nanostructures have attracted increasing attention because of the potential applications, such as sensors, photonic crystals and LEDs. For example, one interesting application is the fabrication of ordered bulk-heterojunction solar cells [6]. This cell consists of interdigitated vertical channels of donor and acceptor materials of width comparable to the exciton diffusion length. Fabrication methods such as nanoimprinting, nanolithography, electrospinning and template [7-10] have been employed to produce organic nanostructures. The template technique has the advantage of being able to create large-area and arrays of structures with control over structural parameters. Anodized aluminium oxide has become one of the most common templates for the preparation of different nanometer-sized structures [11-16]. Under appropriate anodization conditions, long-range-ordered anodic porous alumina with an ideally ordered nanopores arrangement can be obtained [17].

Herein, we report the fabrication of ordered nanostructures employing a template wetting method, which entails the infiltration of a polymeric solution of PFO at room temperature into self-ordered anodic aluminium oxide. By this technique, the obtained nanostructures were studied as entire samples supported on a film and the dimensions were given by the home-made alumina. Nanoporous alumina template was prepared by two-step anodization process of aluminium metal in an acidic solution. The formation of the **β**-phase of the resulting ordered PFO nanopillars was evidenced by the presence of an emission peak at 439 nm and an μ-X-ray diffraction peak centred around 7.0°. We have also investigated the effect of orienting the polymer chains during the infiltration process by Raman spectroscopy.

## Experimental

High purity 99.999% aluminium foils from Goodfellow Cambrigde Ltd. were pre-treated following the procedure reported. The nanoporous alumina templates were prepared by 2-step anodizing process on the aluminium surface [17,18]. The first anodization was performed in 3 wt% phosphoric acid (H_3_PO_4_) at 160 V and -5°C. Subsequently, the sample was immersed into a mixture solution of 0.4 M phosphoric acid (H_3_PO_4_) and 0.2 M chromic acid (H_2_CrO_4_) at 70°C to remove the porous alumina formed during the first anodization. The conditions of the second anodization were the same as the first anodization. In order to enlarge the pore diameter, a pore-widening treatment was carried out in 5 wt% phosphoric acid at 35°C. The depth and pore diameter were adjusted by a second anodization for 3 min and pore-widening treatment for 30 min. Nanoporous alumina templates with pore diameter of 225 nm and pore depth of 500 nm approximately were obtained.

Nanopillars were fabricated under ambient conditions via infiltration of a Poly(9,9-dioctylfluorene) (PFO, M_w_ ~ 58,200 g mol^-1^, Sigma–Aldrich) solution in chloroform (CHCl_3_) (60 mg mL^-1^) into the pores of the alumina template. After 24 h at room temperature, the aluminum was removed by immersion into a solution containing 6.8 g of copper (II) chloride (CuCl_2_), 200 ml of 37% hydrochloric acid (HCl) and 200 ml of deionized water. PFO nanopillars were obtained by the selective dissolution of the alumina template in a 3 M sodium hydroxide (NaOH) aqueous solution at room temperature for 30 min.

All the samples were inspected in environmental scanning electron microscopy (ESEM-FEI Quanta 600). A gold thin layer was deposited on polymer nanostructures in order to avoid deformations upon heating during ESEM observation. Absorption spectra were measured by a double beam UV–Vis Shimadzu spectrophotometer (UV-1700). Luminescence fluorimeter (Aminco-Bowman Series 2), with a system composed by xenon lamps and a wavelength range from 250 to 850 nm, was used for measuring the emission spectrum of polymer samples. The Raman spectra were recorded using excitation laser wavelength 785 nm with a Renishaw Raman Imaging Microscope. Out-of-plane grazing incidence X-ray diffraction (GIXRD) measurements were made using a Bruker-AXS D8-Discover diffractometer, which was operated at 40 kV and 40 mA to generate Cu_Κα_ radiation.

## Results and Discussion

**Figure 1:**
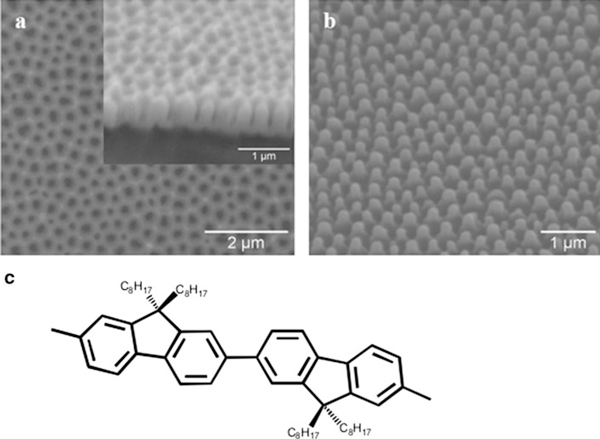
**ESEM images of a top view of the self-ordered alumina template (*Inset*: cross section of the template), b PFO nanopillars after removing the template**. **c** The chemical structure of a segment of a PFO chain in the β-phase conformation. The rotational angle between monomer units is fixed at 180°.

Ordered poly(9,9-dioctylfluorene) nanopillars with β-phase morphology were obtained by replicating from nanoporous alumina via template wetting at room temperature. As explained in the experimental section, the template were fabricated by two-step anodization process in phosphoric acid, yielding templates with an average pore depth of 500 nm and pore diameter of 225 nm (Figure 1a). A scanning electron microscopy (ESEM) image of a PFO nanopillar array acquired after the template dissolution is shown in Figure 1b.

**Figure 2:**
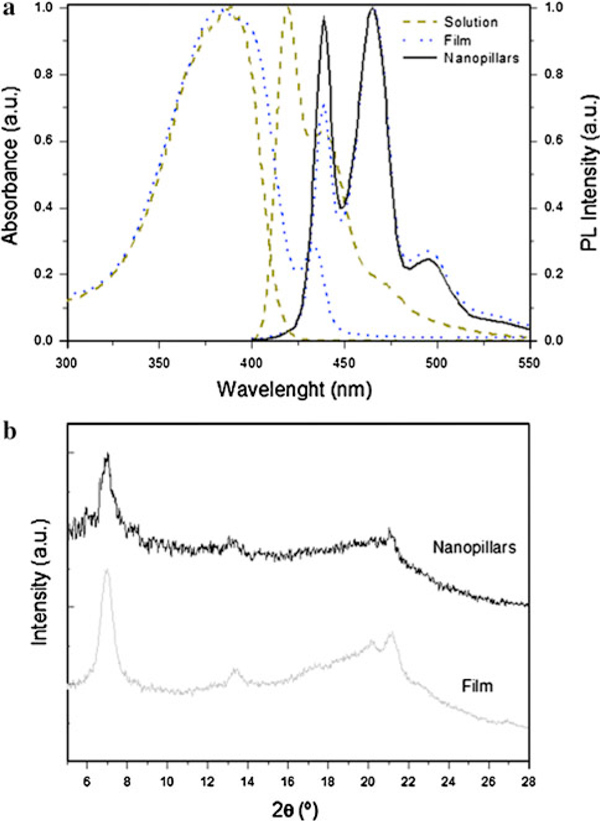
**a UV–Vis absorption and PL spectra of PFO solution, PFO film and PFO nanopillars**. **b** X-ray diffraction patterns of PFO film and PFO nanopillars (*curves* are offset for clarity).

Figure 2 shows the UV–Vis absorbance spectra of a PFO solution in chloroform and a PFO film. The nanopillars spectrum was not possible measured due to the high concentration of the solution that we used in order to fabricate the structures. The solution absorption spectra exhibited a band at 373–395 nm assigned to the S_0_ → S_1_ 0-0 transition of PFO. The film spectrum was red-shifted and broader with a band at 388 nm and a low-energy shoulder at 435 nm characteristic of β-phase PFO [4,19,20].

The photoluminescence (PL) spectra of a PFO solution in chloroform, PFO film and an entire sample of ordered PFO nanopillars are also shown in Figure 2. The solution PL spectrum exhibited a characteristic vibronic progression with peaks located at 418, 437 and 463 nm due to S_0_ → S_1_ 0-0 singlet exciton transition of solution PFO with 0-1, 0-2 and 0-3 vibronic replicas. The PFO film and PFO nanopillar PL spectrum were red-shifted compared to those to the solution, suggesting a narrowed distribution of PFO chain segments with increased conjugation lengths [19]. The nanopillars spectrum exhibited the emission peaks at 439, 464 and 496 nm, which agree with the PL spectrum of β-phase PFO film described in the literature [20]. If we compare the film spectra and the nanostructure spectra, we can observe different intensity in the emission peak at 439 nm. This result seems to indicate that the optical properties of the polymer nanostructure could be affected by the polymer morphology into the nanopores. It could be attributed to ordering or axial alignment of polymer chains into the nanopores of the template during the infiltration process [21,22].

Grazing incidence X-ray diffraction (GIXRD) profiles acquired for a PFO film and PFO nanopillars are shown in Figure 2. The peaks appear at 2θ of 6.98° and 21.16° for PFO film and at 2θ of 7.00° and 21.10° for ordered PFO nanopillars. The peak at 7.0 degrees corresponds to (200) plane, which is in agreement with the XRD peak of the β-phase PFO [20,23].

These results obtained from PL and X-ray measurements reveal that the ordered PFO nanopillars fabricated via template wetting using nanoporous alumina as template are obtained with β-phase morphology.

**Figure 3:**
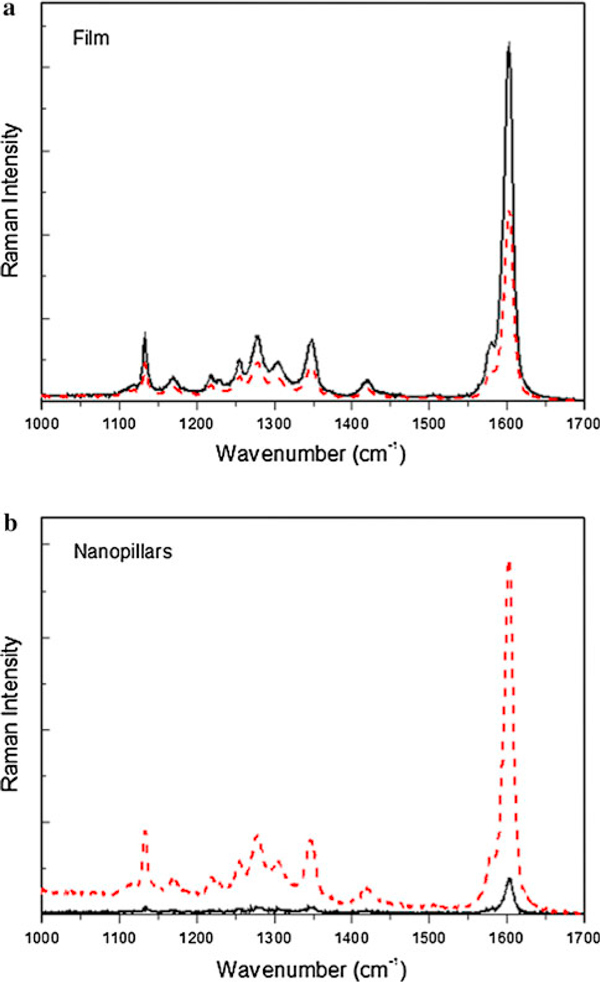
**Raman spectra of PFO film and nanopillars**. The excitation laser is polarized parallel to the polymer chains and detection is polarized parallel (*dashed line*) and perpendicular (*solid line*).

Raman spectroscopy characterization is also performed in order to study the different polymer conformation into the nanoporous template. Raman studies can provide structural information on conjugated polymers, necessary for understanding of optical and electronic properties and the development of devices in which they are used as active layers. We have studied the effect on the Raman spectrum of the polymer chain orientation. The nanostructured samples were excited with the laser beam polarized parallel to the orientation direction of the pillars and the Raman signal measured polarized either parallel or perpendicular to the polymer backbones. Figure 3 shows the Raman spectra in the 1,000–1,700 cm^-1^ range for PFO film and PFO nanopillars. The most intense band is located at 1,604 cm^-1^ and is assigned to the phenyl intra-ring C–C stretch mode [4,24]. We observed that the intensity of the signal polarized parallel to the excitation is higher than that polarized perpendicular when the spectra is acquired for PFO nanopillars, but there are no changes in the intensity of the peaks when the sample is a PFO film. These Raman spectra indicate that the chains of the polymer are mainly parallel to the pillar in the nanostructure, but in the film, the chains are not aligned with respect to the laser beam. These results are in agreement with the PL measurements showed previously, where the emission spectrum is affected by the nanostructure.

## Conclusions

We have presented a fast and easy method based in template wetting for fabricating ordered Poly(9,9-dioctylfluorene) (PFO) nanopillars with β-phase morphology. This method entails infiltration of the polymeric solution of PFO at room temperature into self-ordered anodic aluminium oxide, which is obtained by two-step anodization process of aluminium metal. The formation of the β-phase morphology in the resulting ordered PFO nanopillars was evidenced by the presence of an emission peak at 439 nm and an X-ray diffraction peak centred around 7.0 degrees. The results mean that modifications in the interactions between the polymer chains due to the infiltration in the nanopores could affect the optical properties of the polymer, as is shown in the PL spectra. Raman measurements illustrate that the orientation of the polymer chains in the nanostructures changes compared to the film measurements due to the fabrication process which could be the cause for the differences in the optical properties.

The obtaining of the β-phase morphology in polymer structures could be interesting in photonic and optoelectronic devices due to the optical and electrical properties of this phase. The use of a template, such as nanoporous alumina, allows the preparation of nanopillars with the suitable dimensions in order to be applied in different devices. This work could be extend to other polyfluorene polymers or other conjugated polymer such as derivates of poly(phenylenevinylene).
